# Numerical study of hydrothermal and mass aspects in MHD driven Sisko-nanofluid flow including optimization analysis using response surface method

**DOI:** 10.1038/s41598-023-34960-9

**Published:** 2023-05-15

**Authors:** Xinhua Wang, Ghulam Rasool, Anum Shafiq, Thirupathi Thumma, Qasem M. Al-Mdallal

**Affiliations:** 1 Institute of Intelligent Machinery, Faculty of Materials and Manufacturing, Beijing University of Technology, Beijing, China; 2 Department of Mechanical Engineering, Lebanese American University, Beirut, Lebanon; 3 Department of Mathematics and Statistics, Nanjing University of Information Science & Technology, Nanjing, China; 4 Jiangsu International Joint Laboratory on System Modeling and Data Analysis, Nanjing University of Information Science and Technology, Nanjing, 210044 China; 5 Department of Mathematics, B V Raju Institute of Technology, Narsapur, Medak, Telangana State 502313 India; 6 Department of Mathematical Sciences, UAE University, P.O. Box 15551, Al Ain, United Arab Emirates

**Keywords:** Mechanical engineering, Engineering, Mathematics and computing, Nanoscience and technology, Physics

## Abstract

A steady, incompressible, two-dimensional Sisko-nanofluid flow towards the horizontal direction with no movement in the vertical direction is considered on a stretching/shrinking surface. The power law component (Sisko model) is incorporated under the regime of the porous medium. A magnetic impact is included coming from the MHD in the surface normal direction. In addition, thermal radiation, Brownian diffusion, and thermophoresis are involved in the governing system of equations obtained from the Navier–Stokes model in two-dimensional flow systems. The PDEs are converted into the one-dimensional system using suitable transformations and solved by Galerkin weighted residual method validated with the spectral collocation method. The optimization analysis is performed on heat transfer and skin-friction factors using response surface methodology. The impact of the parameters involved in the model has been testified and is provided in graphical forms. The outcomes indicate that for the values of the porosity factor fluctuating between [0, 2.5], the velocity profile and corresponding boundary layer thickness are lesser towards the maximum value of the parameter, and the results are opposite as the parameter approaches zero. The optimization and sensitivity analysis shows that the transport of heat sensitivity towards thermal radiation, Brownian diffusion, and thermophoresis declined whenever the Nt and Nb increased from low to high and at the medium level of thermal radiation. An increment in the Forchheimer parameter increases the sensitivity of the rate of friction factor, whereas increasing the Sisk-fluid parameter has the reverse effect. Elongation processes like those of pseudopods and bubbles make use of such models. The idea is also widely used in other sectors, such as the textile industry, glass fiber production, cooling baths, paper manufacture, and many more.

## Introduction

Fluid flow analysis on stretching and shrinking surfaces are well-researched in the literature. To be sure, there are two distinct types of stretching phenomena: linear and non-linear. While both have their uses in real-world challenges, the simplicity of linear stretching makes it more trustworthy than non-linear. Stretching surfaces has several significant applications, particularly in boundary layer formulations of fluid problems. Elongation processes like those of pseudopods and bubbles use shrinking/stretching surfaces. The idea is also widely used in other sectors, such as the textile industry, glass fiber production, cooling baths, paper manufacture, and many more. The research of Sakaidis^1^, which is based on boundary layer formulations and the uniform motion of solid surfaces, is an example of relevant literature. One notable piece in this area is Crane’s^2^ description of the boundary layer flow phenomenon, which depends on the stretching of the surface, moves within the plane, and implements the idea of relevant velocity towards a fixed point. Reading Hassanien et al.^3^ explanation of how micro-polar fluids behave when they flow through a stretching surface utilizing the varied temperature distribution on the surface is crucial to getting a firm grasp on this phenomenon. The effect of a magnetic field on the motion of a viscoelastic and electrically conducting fluid based on a stretching surface was studied by Anderson et al.^4^. The reader is directed to^5–10^ for further information on related topics.

Sisko-fluids is a catch-all term for power-law fluids that describes the commonalities amongst, for example, biofluids, greases, cement pastes used in drilling fluids, waterborne fluids, and cement slurries. For his first modelling of siskofluids, Sisko^11^ focused on the fluid dynamics of lubricating greases. With the addition of a superscript (j = 1), the fluid reverts to its Newtonian form, losing its Sisk-property. Therefore, in the power law model, the values (j > 1 or j < 1) play a crucial role. After Khan et al.^12,13^ described the model of nanofluids based on sisko-fluid features, Nadeem, and Akbar^14^ expanded the concept in peristaltic flow by merging the sisko model with the inclined tube surface. Later works incorporated the idea of boundary layer formulations and reported noteworthy discoveries; for instance, Khan and Shehzad^15^ established boundary layer approximations for sisko-fluid models using a stretching surface. It was shown that the wall drag force varies noticeably between linear and non-linear stretching situations. According to the sisko flow model described by Hayat et al.^16^, porous media with blowing or suction capabilities may be used to create the flow. Using the sisko-model and other characteristics including heat production, heat absorption, and thermal radiation, Eid et al.^17^ and Eid and Mahny^18^ developed similar ideas.

Recent years have seen a rise in the importance of MHD (magnetohydrodynamic) research in many practical engineering and industrial uses of fluids. Because MHD has such a dramatic effect on fluid motion and generates different retardational and supporting forces depending on where a magnetic field is applied to a particular surface, it may be used with surprising swiftness to regulate a wide range of conditions. Induction flow meters are a prime example of this since they measure the velocity of a fluid in relation to the direction of a magnetic field's potential difference. Hundreds of researches have been published on the role of MHD in fluid flow issues, and the results have vastly improved. The occurrence of MHD in non-Newtonian fluids is indicative of real-world engineering applications, such as in the production of electricity, the operation of nuclear reactors, the purification of crude oil, and many other areas. Several research have been recorded, indicating that the idea of MHD participation in fluid mechanics has been given considerable attention. Hsiao^19,20^ presented mixed convection analysis of visco-elastic fluid using a stretching velocity coupled with porous medium characteristics, which increases the porosity factor. Sometimes it's necessary to regulate flow by manipulating the fluid's motion, and this is where MHD comes in. The behavior of non-Newtonian fluids was studied by Eid and Mishra^21^ using a porous, non-linear stretching surface. In their investigation of fluid flow, Eid & Mahny^22^ considered the role of multi-scale heat dynamics (MHD) and heat absorption/production. Using boundary layer approximations, Hayat et al.^23^ examined the effect of MHD on a sisko-model-based, three-dimensional flow issue. Researchers have also considered the effects of a number of other models all at once, including the Jeffrey model, the Casson model, the micropolar fluids model, and the sisko-model (see for example^24–27^). Furthermore, the significant improvement in the thermophysical properties of nanofluids subject to various parameters including inclined as well as surface normal MHD, porosity factor, and stretching surfaces has been reported categorically in^28–39^ and cross references cited therein.

The online literature on this subject is a significant source of inspiration for our inquiry. We have studied this fluid flow model in the context of boundary layer formulations on a stretched surface to enhance the thermophysical characteristics of a nanofluid subject to various parameters. The rules on governing heat and mass transfer equations are therefore included in the flow model. The governing non-linear Navier–Stokes equations, which are expressed in two dimensions, have the Darcy-Forchheimer components and MHD together with the power law rule (Sisko model) in the momentum equation and thermal radiation in the energy equation, such that the fluid model responds to a stretching rate at the solid surface due to stretching/shrinking properties of the surface and exhibits the governing equations in PDEs format, which are constructively transformed into ODEs using suitable transformations, and subsequently solved by Galerkin weighted residual method (GWRM) validated with spectral collocation method (SCM). The optimization analysis is performed on heat transfer and skin-friction factors using Response Surface Methodology (RSM). The governing model's significant components are porosity, magnetohydrodynamics (MHD), power law rule (Sisko model), and thermal radiation. To our knowledge, however, there is no research on the subject matter. Therefore, the article can be very handy in different fluid mechanics applications, especially biofluids, greases, cement pastes used in drilling fluids, waterborne fluids, and cement slurries.

## Research questions

1. Using the concept of nanoparticles' molar concentration, what physical model can be realistically adapted to predict boundary layer flows of sisko-fluids?
2. How could a magnetic field in terms of MHD act on fluid flow?
3. Given that the Darcy medium enhances the frictional impact on fluid flow analysis, how can we predict the resulting skin friction?
4. Which scientific approach yields reliable findings?
5. What is the significance of applying Optimization analysis to the current model? How can it be fruitful in analyzing the crucial fluctuations in heat transfer rate and wall drag force?
6. How can we enhance the nanofluidic medium's thermal performance?"
7. How can we reduce the magnitude of the forces exerted by friction at the surface?
8. How does the suggested nanofluid flow issue manifest hydrothermally?

### Governing equations and physical model

**Figure 1:**
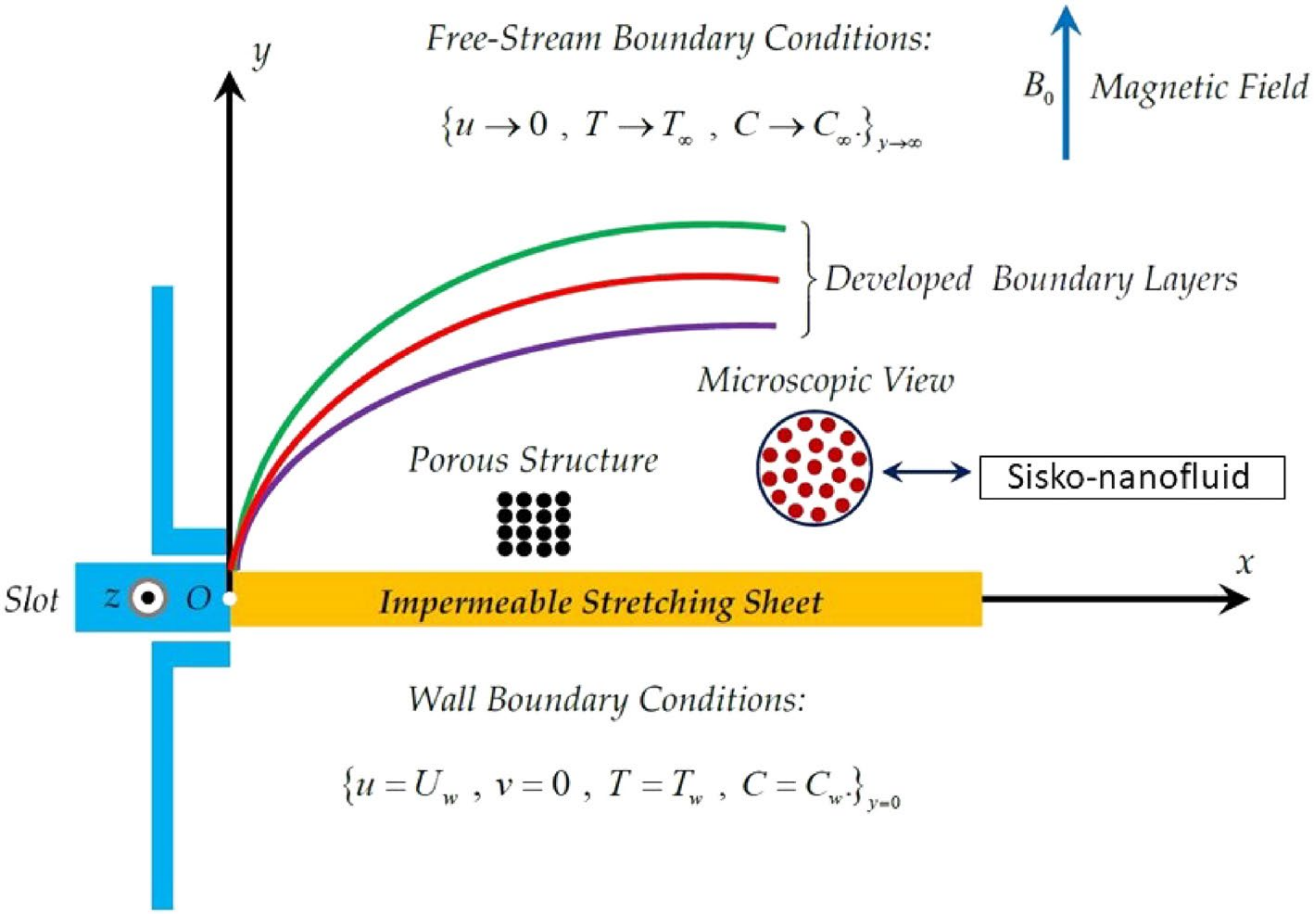
Geometry of the problem.

The present study incorporates Buongiorno’s model utilizing the Sisko-fluid properties adopting a laminar, steady, two-dimensional flow in the positive plane of xy-coordinate system subject to zero movement in vertical direction. The only fluid motion is taken in the x-direction for both shrinking and stretching cases of the surface depending on the stretching/shrinking rate of the surface taken as $u={u}_{w}$. The power law component is represented by $j, j\ne 1$. For $j=1$ the model reverts to the simple Newtonian models. A magnetic impact is included in terms of ${B}_{0}$ coming from the MHD in the surface normal direction, as shown in the geometry. The temperature and concentration are taken as constant values $({T}_{w}, {C}_{w})$, respectively, at the solid surface, whereas $(T\_\infty , C\_\infty )$ represent their ambient states. The system represents proper boundary layer formulations for both ways (stretching or shrinking). The geometry of the stretching surface has been described in Fig. 1. Mentioning all the boundary conditions.

The detailed study of available published literature regarding nanofluid flow shows that porous structures are highly applicable in many industrial applications (see, for example^23,27,29^). Hence, in the present model, we have emphasized the development of fluid flow using a porous medium. Referring to Khan et al.^40^ and Prasannakumara et al.^41^, we can write the complete governing model as follows:

1 $$\frac{\partial u}{{\partial x}} + \frac{\partial v}{{\partial y}} = 0,$$

2 $$u\frac{\partial u}{{\partial x}} + v\frac{\partial u}{{\partial y}} = \frac{m}{\rho }\frac{{\partial^{2} u}}{{\partial y^{2} }} - \sigma \frac{{B_{0}^{2} }}{\rho }u - \frac{n}{\rho }\frac{\partial }{\partial y}\left( {\frac{\partial u}{{\partial y}}} \right)^{j} - \frac{{\mu_{f} }}{{\rho_{f} }}\left( \frac{u}{K} \right) - \frac{{C_{b} }}{x\sqrt K }\left( {u^{2} } \right),$$

3 $$u\frac{\partial T}{{\partial x }} + v\frac{\partial T}{{\partial y }} = \alpha \frac{{\partial^{2} T}}{{\partial y^{2} }} + \frac{{\left( {\rho C_{P} } \right)_{np} }}{{\left( {\rho C_{P} } \right)_{f} }} \frac{\partial T}{{\partial y}}\left( {D_{B} \frac{\partial C}{{\partial y}} + \frac{{D_{T} }}{{T_{\infty } }}\frac{\partial T}{{\partial y}}} \right) - \frac{1}{{\left( {\rho c} \right)_{f} }}\frac{{\partial q_{r} }}{\partial y} ,$$

4 $$u\frac{\partial C}{{\partial x}} + v\frac{\partial C}{{\partial y}} = \frac{{D_{Th} }}{{T_{\infty } }} \frac{{\partial^{2} T}}{{\partial y^{2} }} + D_{Br} \frac{{\partial^{2} C}}{{\partial y^{2} }},$$

The relevant governing boundary conditions (see for example, Mahmood et al.^42^), are listed below:

5 $$\left\{ {u = \varepsilon u_{w} , \, v = 0, \, T,C = T_{w} ,C_{w} {\text{ at}} y = 0} \right\},$$

6 $$\left\{ {u \to 0, \, T,C \to T_{\infty } , C_{\infty } \, {\text{as}} y \to \infty } \right\}.$$

The following transformations, highly suitable to power law models, are introduced in the governing PDEs to resolve them into ODEs for subsequent solutions via numerical scheme (see, for example^23,39^):

7 $$\left\{ {\eta = \frac{y}{x}{\text{Re}}_{n}^{{\frac{1}{j + 1}}} , \, f = \frac{\psi }{{u_{w} x{\text{Re}}_{n}^{{ - \left( {\frac{1}{j + 1}} \right)}} }}, \, \theta = \frac{{T - T_{\infty } }}{{T_{w} - T_{\infty } }}, \, \phi = \frac{{C - C_{\infty } }}{{C_{w} - C_{\infty } }}, \, } \right\}.$$

Accordingly, the following ODEs are obtained together with the converted boundary conditions given in Eqs. (8)–(12), respectively.

8 $$\Lambda f^{\prime\prime\prime} + j( - f^{\prime\prime})^{j - 1} f^{\prime\prime\prime} + \frac{2j}{{j + 1}}ff^{\prime\prime} - \left( {1 + Fr)f^{{\prime}{2}} } \right) - M_{1} f^{\prime} - \lambda f^{\prime} = 0,$$

9 $$\left( {1 + \frac{4}{3}Rd} \right)\theta^{\prime\prime} + \Pr \left( {\frac{2j}{{j + 1}}} \right)f\theta^{\prime} + Nb\theta^{\prime}\phi^{\prime} + Nt\theta^{{\prime}{2}} = 0,$$

10 $$Le\Pr \left( {\frac{2j}{{j + 1}}} \right)f\phi^{\prime\prime} + \frac{Nt}{{Nb}}\theta^{\prime\prime} = 0,$$

11 $$\left\{ {f\left( \eta \right) = 0, \, f^{\prime}\left( \eta \right) = \varepsilon , \, \theta \left( \eta \right) = 1, \, \phi \left( \eta \right) = 1{\text{, at}} \eta = 0} \right\},$$

12 $$\left\{ {f^{\prime}\left( \eta \right) \to 0, \, \theta \left( \eta \right) \to 0, \, \phi \left( \eta \right) \to 0 \, {\text{as}} \eta \to \infty } \right\}.$$

A significant number of parameters are involved in the systems (1)–(12); therefore, a complete nomenclature of the parameters is necessarily provided below in the Table 1.

**Table 1:** A summarizing list of the pertinent parameters with their significance.

Parameters	Symbols	Expressions
Magnetic parameter	$M_{1}$	$\frac{{\sigma_{f} B_{0}^{2} }}{\rho C}$
Prandtl number	$\Pr$	$\frac{{xu_{w} }}{\alpha }{\text{Re}}_{n}^{{\frac{ - 2}{{j + 1}}}}$
Sisk-fluid parameter	$\Lambda$	$\Lambda = \frac{{{\text{Re}}_{n}^{{\frac{2}{j + 1}}} }}{{{\text{Re}}_{m} }}$
Thermophoresis parameter	$N_{Th} (or\,Nt)$	$\frac{{\tau D_{Th} \left( {T_{w} - T_{\infty } } \right)}}{{\upsilon_{{}} T_{\infty } }}$
Brownian motion parameter	$N_{Br} \,(or\,Nb)$	$\frac{{ \tau D_{Br} C_{\infty } }}{\upsilon }$
Lewis number	Le	$\frac{\alpha }{{D_{Br} }}$
Forchheimer number	Fr	$\frac{{C_{b} }}{\sqrt K }$
Porosity parameter	$\lambda$	$\frac{\upsilon }{\varepsilon K}$
Radiation parameter	$Rd$	$\frac{{16\sigma *T_{\infty }^{3} }}{3kk*}$
Local Reynolds number	${\text{Re}}_{m}$	$\frac{{\rho xu_{w} }}{m}$

Significantly, the dimensionless expressions of the total viscous frictional factor $C_{fr }$ (i.e., the skin friction coefficient) and the wall thermal transfer rate $Nu_{x}$ (i.e., Nusselt number) are given locally by:

13 $$C_{fr } = \tau_{w} \frac{1}{{\rho u_{w}^{2} }},$$

14 $$Nu_{x } = \frac{{xq_{w} }}{{k\left( {T_{w} - T_{\infty } } \right)}},$$

Moreover, the involved shear stress components $\left( {\tau_{wr } ,\tau_{w\varphi } } \right)$, along with the heat flux, $q_{T}$ have the following expressions:

15 $$\tau_{w} = \left( {m\frac{\partial u}{{\partial y}} - n\left( { - \frac{\partial u}{{\partial y}}} \right)^{j} } \right)_{y = 0} ,$$

16 $$q_{w } = - k\left( {\frac{\partial T}{{\partial y}}} \right)_{y = 0} + \left( {q_{r} } \right)_{w} ,$$

The finaly simplified version of system (15)–(16) is given in (17)–(18), as follows:

17 $${\text{Re}}_{n}^{{\frac{1}{j + 1}}} C_{f } = \{ \Lambda f^{\prime\prime} - \left( { - f^{\prime\prime}} \right)^{j} \}_{\eta = 0} ,$$

18 $${\text{Re}}_{n}^{{\frac{ - 1}{{j + 1}}}} Nu = - \left\{ {\left( {\frac{4}{3}Rd + 1} \right)\theta^{\prime}} \right\}_{\eta = 0} ,$$

### Solution methodology and comparative analysis

GWRM, also known as Galerkin weighted residual method, is used to solve the final ODEs. The method can be called a modification case of the Least Squares method. It prioritizes the approximating function derivative over the residual function derivative for the relevant unknown ${a}_{i}$. The weight function can be reproduced as follows:19

GWRM is an effective technique for finding BVP solutions for the following reasons:

1. In the governing differential equations, the initial guesses are assumed to be combinations of trial functions with unknown coefficients.
2. These solutions are incorporated in the equations which contain residuals.
3. The errors are restricted to a low ratio. In addition,
  - The easiness of handling BVPs,
  - High precision, fast convergence, and efficiency,
  - The minimized range within 0 to $\infty$, are excellent feature of this method.

The approximate solutions are sought out as follows:

$$f\left(x\right)= -L\left(\chi \left(x\right)\right), in {D}_{0}$$

where $\chi \left(x\right)$ is an unknown dependent variable corresponding to the function $f(x)$ within the domain ${D}_{0}$ with the operator L. The approximation of the solution comes from the sum of the initial value with the summation of approximated results for any index $1\le k\le n$. In general, one can write,

$${\int }_{D}R\left(x\right){\chi }_{k}\left(x\right)dx=0, k=\mathrm{0,1},2,\dots , n$$

Table 2 presents data that explains the arguments and corresponding coefficient values at the respective argument. At approximately 30th argument, the coefficient becomes negligibly small.

**Table 2:** Arguments and corresponding coefficients.

Arguments ${x}_{k}$	Coefficients ${B}_{k}$
0.137793	0.308441116
0.729455	0.401119929
1.80834	0.218068288
3.40143	0.062087456
5.5525	0.009501517
8.33015	0.000753008
11.8438	0.000028259
16.2793	4.24931 × 10^–7^
21.9966	1.83956 × 10^–9^
29.9207	9.91183 × 10^–13^

**Figure 2:**
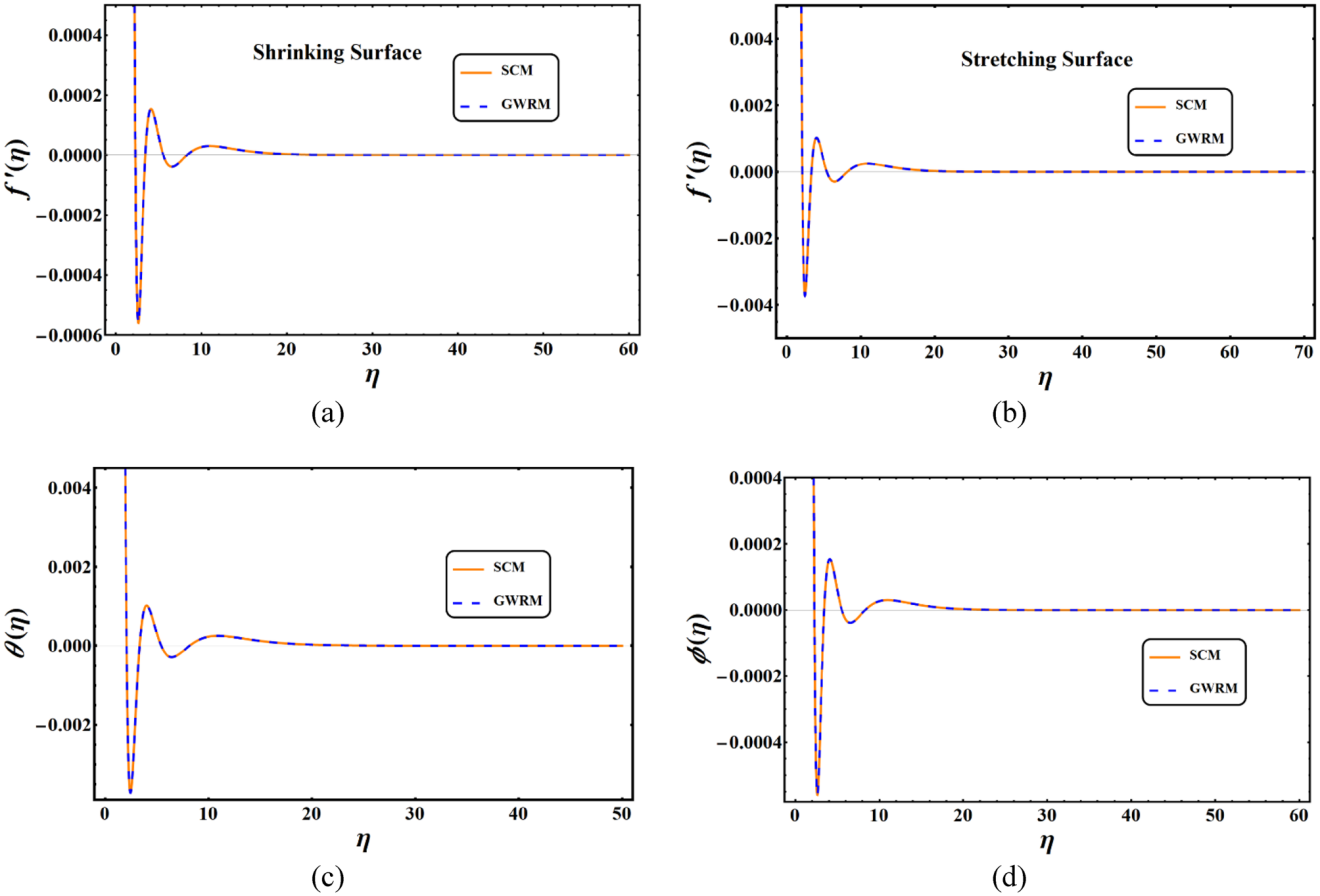
Comparative analysis of SCM and GWRM for different cases.

A comparative analysis was performed for the spectral collocation method (SCM) and Galerkin weighted residual method (GWRM). Figure 2a,b represent the comparative analysis of velocity profiles for shrinking and stretching surfaces, respectively. An elegant agreement has been found in both cases. Figure 2c,d represent the comparative analysis of SCM and GWRM for temperature and concentration profiles, respectively.

## Outcomes and discussions

**Figure 3:**
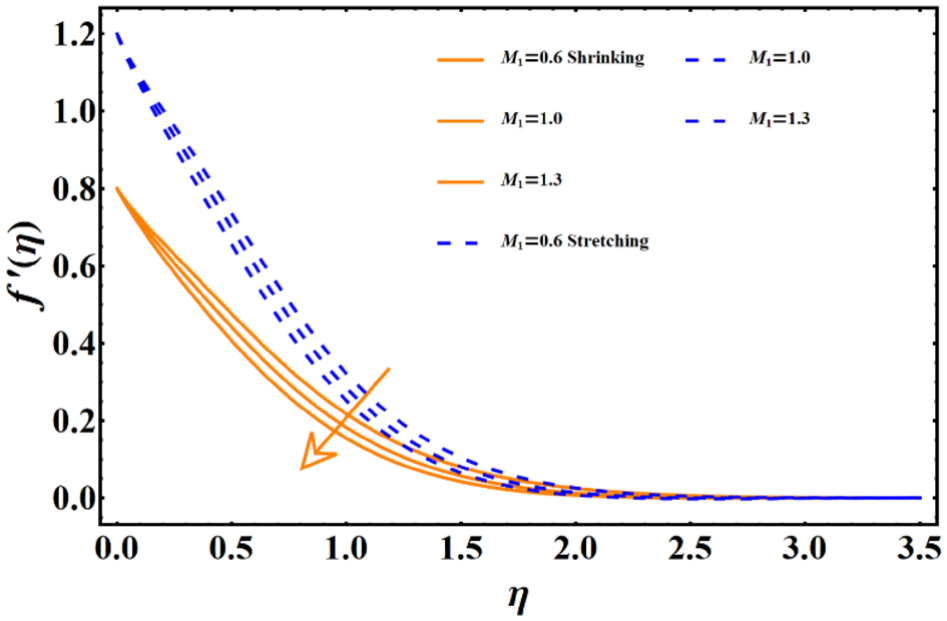
The impact of magnetic parameter on fluid movement.

**Figure 4:**
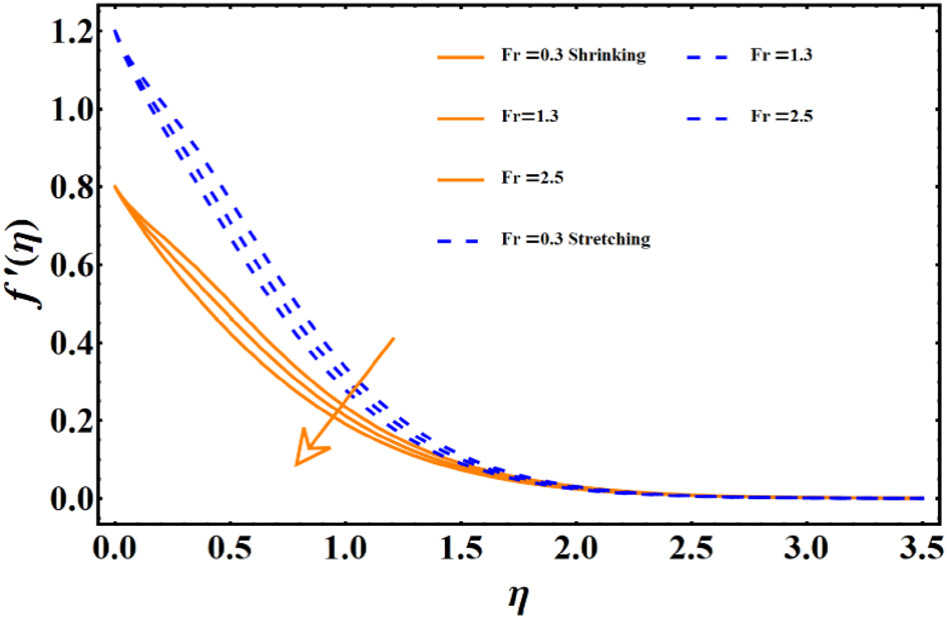
The impact of Forchheimer number on fluid movement.

**Figure 5:**
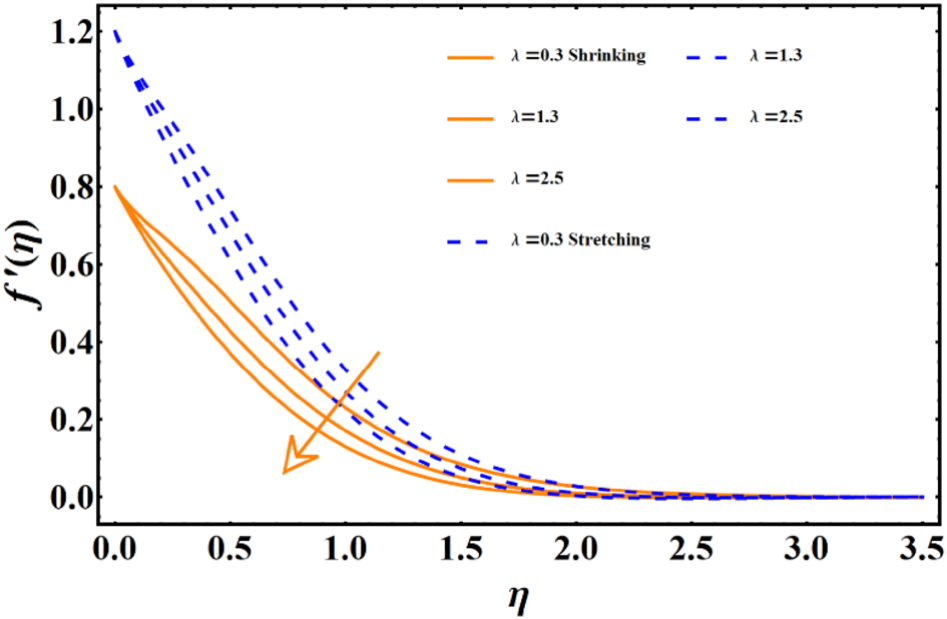
The impact of Porosity number on fluid movement.

**Figure 6:**
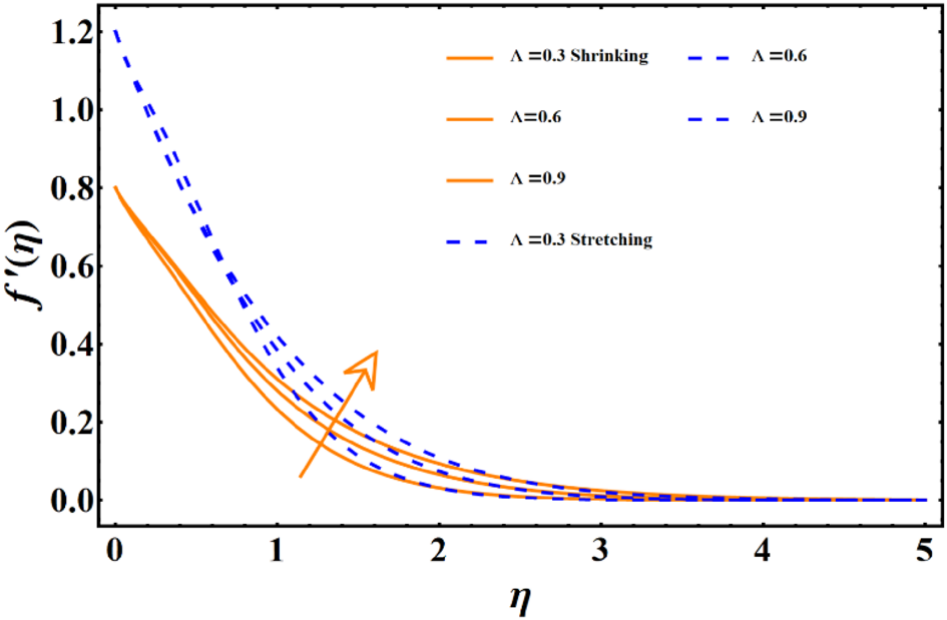
Impact of material parameter on fluid movement.

The impact of various parameters named as magnetic field, thermal radiation, Forchheimer number, porosity number, Sisko-fluid parameter, and power law index represented here with notation j, Brownian diffusion, thermophoresis & Prandtl number on all the three main profiles of fluid flow are analyzed by finding numerical data which is subsequently plotted in graphical forms. A dual analysis is performed within the same frame of reference for each parameter for shrinking and stretching surfaces. Solid lines in the graph represent shrinking surfaces, while dashed lines represent the stretching surface. Specifically, the diagram in Fig. 3 depicts the importance of magnetic field parameters and the consequences of incremental trends in its values on the flow profile. In both cases, the profile undergoes the same declining trend for shrinking and stretching. The velocity profile and the corresponding boundary layer thickness are much smaller for larger values of the parameters. In contrast, when the values of the parameters are closer to zero or approaching zero, the profile shows more thickness and a higher temperature profile. The rise of a Lorentz force due to magnetic parameters is solely responsible for this case. The surface normal direction of the field lines directly interrupts the fluid movement, and therefore, the profile shows a decline when the magnetic field impact is enhanced. The influence of the involvement of porous medium in fluid flow analysis generally appears in terms of the Forchheimer and porosity numbers. Figure 4 reflects the traits of the velocity profile governed by the elevated values of the Forchheimer number, which consequently results in an inevitable decline in fluid flow. Physically, the inertial influence occurring due to the Fr results in the deterioration of the fluid velocity. Similar trends appear in the case of the porosity number given in Fig. 5. For the values fluctuating between $0\le \lambda \le 2.5$, the given profile and corresponding boundary layer thickness are lesser towards $\lambda =2.5,$ and the results are opposite as $\lambda =0$ is approached. This trend appears due to the enhancement of frictional force by adding the porosity factor. At $\lambda =0$, the fluid flow medium returns to a simple medium having no porosity and no additional frictional force. The impact of the material parameter, also known as the sisko-fluid parameter, on the velocity distribution is given in Fig. 6, which reflects the rise in velocity profile for larger parameter values. Looking back, the constituency term of the material parameter is shown in Table 1, the material parameter (Sisko-fluid parameter) comprises the consistency index and the shear rate viscosity. The inverse relations depict that a rise in material parameter diminishes the viscosity, and therefore, the fluid movement catches a jump.

**Figure 7:**
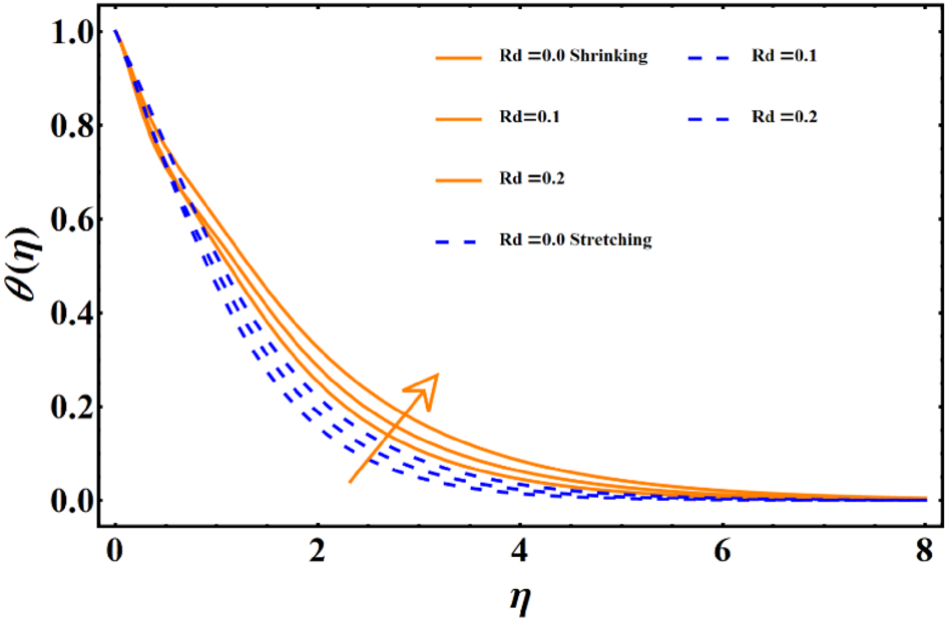
The impact of thermal radiation on temperature distribution.

**Figure 8:**
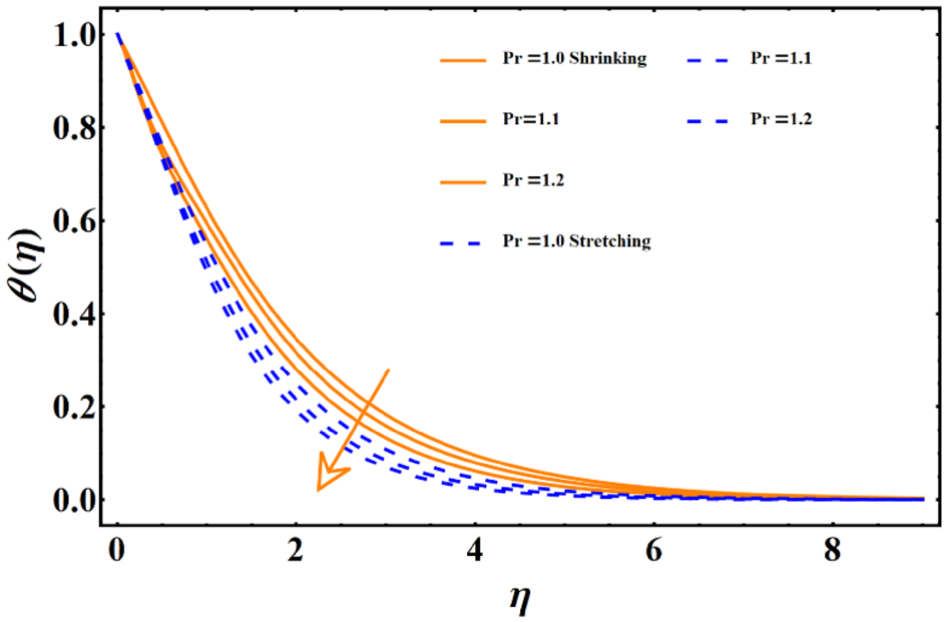
The impact of Prandtl number on temperature distribution.

**Figure 9:**
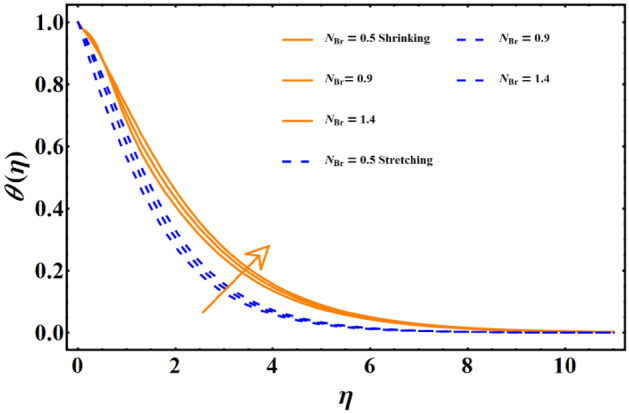
The influence of Brownian diffusion on the temperature distribution.

**Figure 10:**
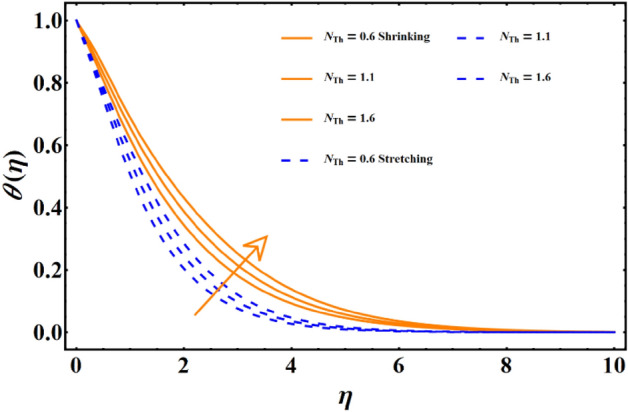
The influence of thermophoresis on the temperature distribution.

**Figure 11:**
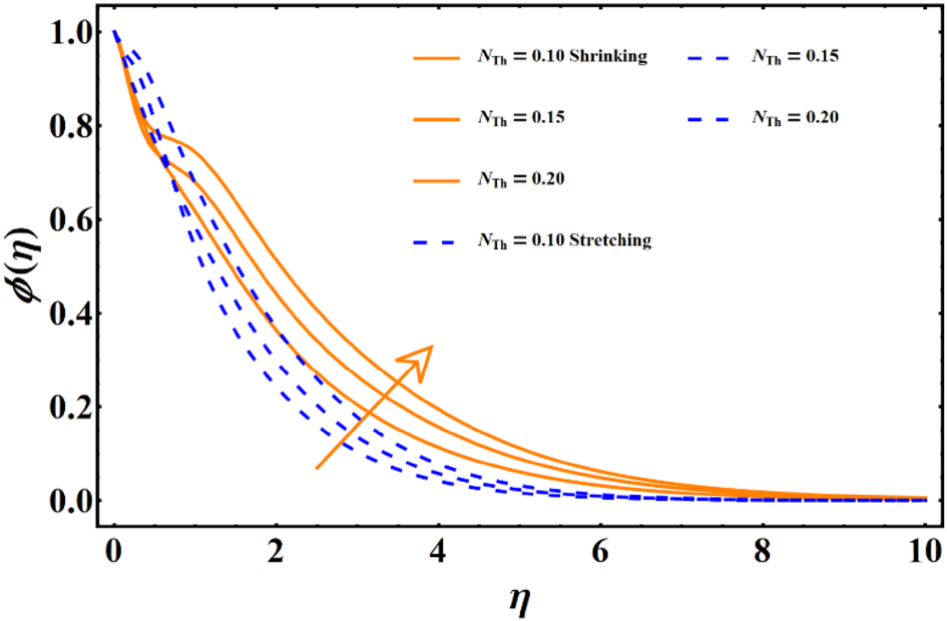
The influence of thermophoresis on the concentration distribution.

**Figure 12:**
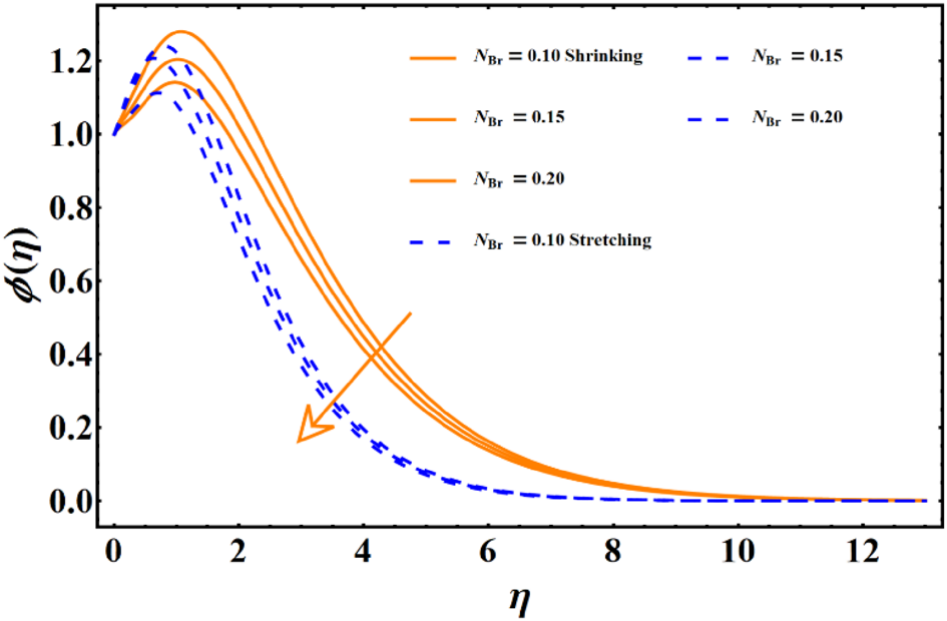
The influence of Brownian diffusion on concentration distribution.

**Figure 13:**
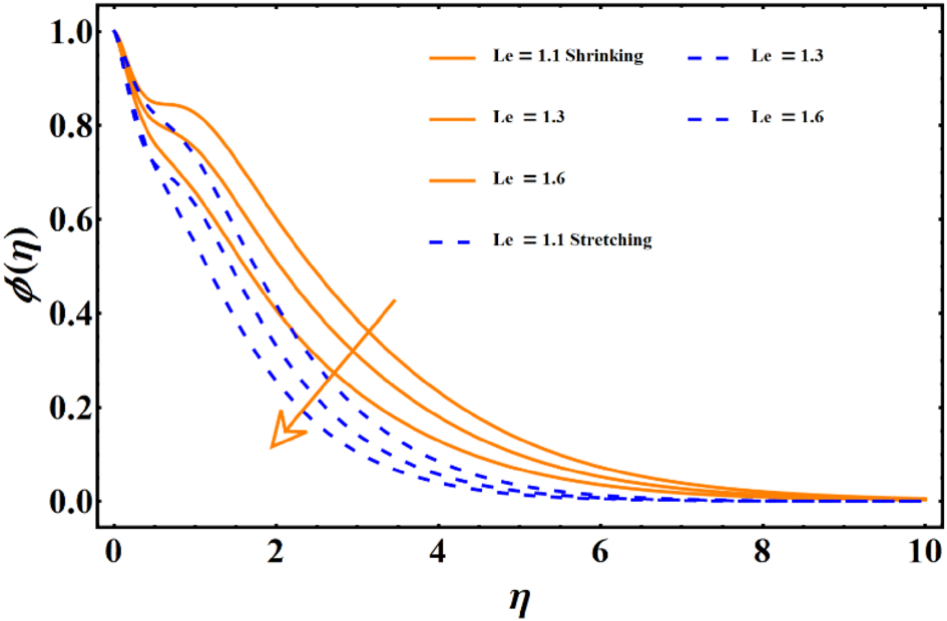
The influence of Lewis number on the concentration distribution.

Figure 7 reflects the trends in temperature distribution uprising due to the thermal radiation factor involved in the model subject to Rossland’s radiative process. For both cases, stretching & shrinking, a significant rise is noticed in thermal distribution because thermal radiation predominates significantly over the fluid's thermal conduction property, resulting in increased temperature distribution and corresponding thickness of the boundary layer. Figure 8 exhibits the development in the temperature distribution due to elevated values of the generalized Prandtl number. Since a higher Prandtl number appears to keep the lower thermal conductivity of the material under consideration, known from the constituency term of the Prandtl number given in Table 1, therefore, the conduction declines. Ultimately the situation gives rise to the boundary layer thickness, and a declining trend is noticed in the temperature distribution profile. Figure 9 reflects the temperature distribution profile for rising values of the Brownian diffusion parameter. The activation of Brownian diffusion creates a hassle for the nanoparticles to find their colder region, thus colliding more rapidly and resulting in the rise of the thermal state. The boundary layer thickness diminishes accordingly. Figure 10 reflects the trends of temperature distribution for elevated values of the thermophoresis parameter. The temperature distribution results in an inevitable rise due to the incident in predictive force activated within the fluid, resulting in rapid collisions and a heat transfer process. For both the cases, shrinking and stretching, we have noticed that the concentration of the particles shows particular rising behavior for the range $0\le {N}_{Th}\le 1,$ but the impact of thermophoresis becomes negligible close to 0, as portrayed in Fig. 11; however, the results for both cases are opposite for the Brownian diffusion parameter for the range of parametric values for the concentration profile. For both shrinking & stretching, we notice the decline in concentration profile is prominent, even close to the lowest possible value for the Brownian diffusion parameter given in Fig. 12. The impact of Lewis number on the concentration profile is portrayed in Fig. 13 reflects a certain decline in the concentration of the nanoparticles. Since the constituency of Lewis number is given in Table 1. Shows a ratio of thermal diffusion & species diffusion rate within the given geometry. Thus, incremental values of the Lewis number reduce the thickness of the thermal boundary layer, resulting in a cumulative trend in the concentration profile. Numerical findings of skin friction and Nusselt number are present in Table 3. The corresponding value for SF and Nu is calculated for different parameters for each altered value. Nusselt shows an augment for higher values of radiation factor and Brownian diffusion. Skin friction rises for porosity factors.

**Table 3:** Numerical results for skin-friction and Nusselt number.

Skin friction $- F^{\prime\prime}\left( 0 \right)$				Nusselt number $-{\theta }^{^{\prime}}\left(0\right)$				
$\Lambda$	Fr	$\lambda$	$R{e}_{r}^\frac{1}{2}{C}_{fr}$	R_d_	N_Th_	N_Br_	Pr	− Nu
0.1	0.1	0.2	0.12605	0.2	0.3	0.1	1.0	1.32567
0.2			0.39891	0.3				1.32681
0.3			0.68185	0.4				1.32882
0.4			0.79963	0.5				1.33675
0.5	0.0	0.2	0.61819	0.5	0.1	0.3	1.0	1.66667
	0.2		0.77872		0.5			1.66671
	0.4		0.86195		1.0			1.66682
	0.6		0.13848		1.5			1.66695
	0.8		0.29783		2.0			1.66702
0.5	0.3	0.1	0.93782		0.5	0.1	1.0	1.53457
		0.3	1.12772			1.0		1.63651
		0.5	1.22527			2.0		1.65672
		0.7	1.24365			3.0		1.66897
		0.9	1.31629			4.0		1.67719
						0.2	1.0	0.99988
							1.2	0.99996
							1.4	1.00036
							1.6	1.00047
							1.8	1.00052

### Optimization analysis

To determine the optimal response of heat transfer rate and the skin friction coefficients for the efficiency of specific components, a recently developed statistical method known as Response Surface Methodology (RSM) is used. The current simulation is dependent on thermal radiation, Brownian motion, and thermophoresis variables for the local Nusselt number, and the simulation is dependent on the magnetic field parameter, Sisk-fluid parameter, and Forchheimer parameter for the skin friction coefficient, respectively. The simulation process is carried out within the allowed range of these parameters. These factors are considered when determining the heat transfer rate as $0.1 \le Rd \le 0.3$, $0.2 \le Nb \le 0.4$&$0.1 \le Nt \le 0.3$ and for friction factor as $0.1 \le M_{1} \le 0.3$, $0.1 \le \Lambda \le 0.5$, $0.2 \le Fr \le 0.6$. To enforce the suggested second-order model, we use the CCD's in-built linear, quadratic, and interaction terms with low, medium, and high values, as the face-centered design method recommended. These ranges with their corresponding levels for the components $\left( {Rd,Nb,Nt} \right)$ with coded symbols (A, B, C) and $\left( {M_{1} ,\Lambda ,Fr} \right)$ coded symbols (A, B, C) used for the response of the Nusselt number and the skin friction coefficient are arrayed through Tables 4 and 5, respectively. The distribution of the corresponding answers obtained from 20 separate runs employing the various parameters is shown in Tables 6 and 7, which are given one after the other. In addition, the multivariate models for the response function in terms of the relevant elements are provided by the entire quadratic polynomial as

$$\begin{gathered} Nusselt\, number = 0.861304 - ~0.006460~~*Rd{\text{ }} + ~0.135520~*Nb - ~0.005830~*Nt - ~0.001009~*Rd^{2} \hfill \\ - ~0.004709*Nb^{2} {\text{ }} + ~0.000241~~*Nt^{2} - ~0.006925*Rd*Nb{\text{ }} + ~0.001025*Rd*Nt - ~0.000675~*Nb*Nt \hfill \\ \end{gathered}$$

$$\begin{gathered} Skin \, friction = 0.282955 - 0.000680 *M_{1} \, + 0.045290 *\Lambda \, + 0.088680*Fr + 0.000164 *M_{1}^{2} \, \hfill \\ - 0.000286 *\Lambda^{2} \, - 0.001936*Fr^{2} - 0.000200*M_{1} *\Lambda - 0.000450 *M_{1} *Fr \, + 0.013825 *\Lambda *Fr \hfill \\ \end{gathered}$$

**Table 4:** Range of independent factors and their levels to assess Nusselt number.

Codes	Parameter	Levels		
		Low_(− 1)	Middle_(0)	High_(+ 1)
$A$	$0.1 \le Rd \le 0.3$	0.1	0.2	0.3
$B$	$0.2 \le Nb \le 0.4$	0.2	0.3	0.4
$C$	$0.1 \le Nt \le 0.3$	0.1	0.2	0.3

**Table 5:** Range of independent factors and their levels to assess Skin friction.

Codes	Parameter	Levels		
		Low_(– 1)	Middle_(0)	High_(+ 1)
$A$	$0.1 \le M_{1} \le 0.3$	0.1	0.2	0.3
$B$	$0.1 \le \Lambda \le 0.5$	0.1	0.3	0.5
$C$	$0.2 \le Fr \le 0.6$	0.2	0.4	0.6

**Table 6:** Experimentation design for the Nusselt number.

Runs	Coded values			Real values			Response
	Low	Middle	High	$Rd$	$Nb$	$Nt$	Nusselt number
1	− 1	− 1	− 1	0.10	0.20	0.10	0.7256
2	1	− 1	− 1	0.30	0.20	0.10	0.7252
3	− 1	1	− 1	0.10	0.40	0.10	1.0123
4	1	1	− 1	0.30	0.40	0.10	0.9835
5	− 1	− 1	1	0.10	0.20	0.30	0.7136
6	1	− 1	1	0.30	0.20	0.30	0.7166
7	− 1	1	1	0.10	0.40	0.30	0.9969
8	1	1	1	0.30	0.40	0.30	0.9729
9	− 1	0	0	0.10	0.30	0.20	0.8675
10	1	0	0	0.30	0.30	0.20	0.8531
11	0	− 1	0	0.20	0.20	0.20	0.7213
12	0	1	0	0.20	0.40	0.20	0.9919
13	0	0	− 1	0.20	0.30	0.10	0.8674
14	0	0	1	0.20	0.30	0.30	0.8557
15	0	0	0	0.20	0.30	0.20	0.8613
16	0	0	0	0.20	0.30	0.20	0.8613
17	0	0	0	0.20	0.30	0.20	0.8613
18	0	0	0	0.20	0.30	0.20	0.8613
19	0	0	0	0.20	0.30	0.20	0.8613
20	0	0	0	0.20	0.30	0.20	0.8613

**Table 7:** Experimentation design for the skin friction.

Runs	Coded values			Real values			Response
	Low	Middle	High	$M_{1}$	$\Lambda$	$Fr$	Skin friction
1	− 1	− 1	− 1	0.1	0.1	0.2	0.1611
2	1	− 1	− 1	0.3	0.1	0.2	0.1606
3	− 1	1	− 1	0.1	0.5	0.2	0.224
4	1	1	− 1	0.3	0.5	0.2	0.2232
5	− 1	− 1	1	0.1	0.1	0.6	0.3114
6	1	− 1	1	0.3	0.1	0.6	0.3096
7	− 1	1	1	0.1	0.5	0.6	0.4301
8	1	1	1	0.3	0.5	0.6	0.427
9	− 1	0	0	0.1	0.3	0.4	0.2835
10	1	0	0	0.3	0.3	0.4	0.2829
11	0	− 1	0	0.2	0.1	0.4	0.2371
12	0	1	0	0.2	0.5	0.4	0.3284
13	0	0	− 1	0.2	0.3	0.2	0.1923
14	0	0	1	0.2	0.3	0.6	0.3699
15	0	0	0	0.2	0.3	0.4	0.2829
16	0	0	0	0.2	0.3	0.4	0.2829
17	0	0	0	0.2	0.3	0.4	0.2829
18	0	0	0	0.2	0.3	0.4	0.2829
19	0	0	0	0.2	0.3	0.4	0.2829
20	0	0	0	0.2	0.3	0.4	0.2829

We then reach the following response functions by removing the insignificant components from the response function and calculating the regression coefficients.

$$\begin{aligned} & Nusselt \, number = 0.861304 \, - 0.006460 *Rd \, + 0.135520 *Nb \, - 0.005830 *Nt \, - 0.001009 *Rd^{2} \\ & - 0.004709*Nb^{2} \, - 0.006925*Rd*Nb \, + 0.001025*Rd*Nt \, - 0.000675 *Nb*Nt \\ \end{aligned}$$

$$\begin{aligned} & Skin\;friction = 0.282955 - 0.000680 *M_{1} \, + 0.045290 *\Lambda \, + 0.088680*Fr \\ & - 0.001936*Fr^{2} - 0.000450 *M_{1} *Fr \, + 0.013825 *\Lambda *Fr \\ \end{aligned}$$

The regression coefficients are obtained via the Design of Experiments with RSM.

### Validity of the model

A statistical instrument that is reliable. Analysis of Variance (ANOVA) is used to assess the regression models and the different statistical tests with F-values, p-values, lack of fitting, error, and total error for the simulated outcomes of Nusselt number and the skin friction coefficient is deployed via Tables 8 and 9. The results of the tests on the suggested data set are produced for either a significance level of 5% or a degree of confidence of 95%. The previous research demonstrates that to get a superior accuracy model, it is always preferable to choose the F-values that are more than one and the p-values that are lower than 0.05. In addition, based on the computed result that is displayed in Table 8 (which was obtained from the ANOVA), and depending on the range of the factors with respective p-values, the proposed model of the Nusselt number is eliminated along with the interaction terms, and the expression for this is shown in the body of the text.

**Table 8:** Analysis of variance for Nusselt number.

Source	DF	Adj SS	Adj MS	F-value	P-value	Coefficients
Model	9	0.184946	0.020550	118,354.49	0.000	0.861304
Linear	3	0.184414	0.061471	354,041.75	0.000	
A	1	0.000417	0.000417	2403.52	0.000	− 0.006460
B	1	0.183657	0.183657	1,057,764.15	0.000	0.135520
C	1	0.000340	0.000340	1957.58	0.000	− 0.005830
Square	3	0.000137	0.000046	262.07	0.000	
A*A	1	0.000003	0.000003	16.13	0.002	− 0.001009
B*B	1	0.000061	0.000061	351.23	0.000	− 0.004709
C*C	1	0.000000	0.000000	0.92	**0.360**	0.000241
2-way interaction	3	0.000396	0.000132	759.66	0.000	
A*B	1	0.000384	0.000384	2209.59	0.000	− 0.006925
A*C	1	0.000008	0.000008	48.41	0.000	0.001025
B*C	1	0.000004	0.000004	20.99	0.001	− 0.000675
Error	10	0.000002	0.000000			
Lack-of-fit	5	0.000002	0.000000	*	*	
Pure error	5	0.000000	0.000000			
Total	19	0.184948				

S	R-sq	R-sq (adj)	R-sq (pred)			
Model summary						
0.0000546	100.00%	100.00%	99.99%			

Significant values are in bold.

**Table 9:** Analysis of variance for skin friction.

Source	DF	Adj SS	Adj MS	F-value	*P*-value	Coefficients
Model	9	0.100709	0.011190	123,323.47	0.000	0.282955
Linear	3	0.099158	0.033053	364,271.04	0.000	
A	1	0.000005	0.000005	50.96	0.000	− 0.000680
B	1	0.020512	0.020512	226,059.76	0.000	0.045290
C	1	0.078641	0.078641	866,702.40	0.000	0.088680
Square	3	0.000020	0.000007	75.08	0.000	
A*A	1	0.000000	0.000000	0.81	**0.389**	0.000164
B*B	1	0.000000	0.000000	2.49	**0.146**	− 0.000286
C*C	1	0.000010	0.000010	113.64	0.000	− 0.001936
2-way interaction	3	0.001531	0.000510	5624.30	0.000	
A*B	1	0.000000	0.000000	3.53	**0.090**	− 0.000200
A*C	1	0.000002	0.000002	17.85	0.002	− 0.000450
B*C	1	0.001529	0.001529	16,851.51	0.000	0.013825
Error	10	0.000001	0.000000			
Lack-of-fit	5	0.000001	0.000000	*	*	
Pure error	5	0.000000	0.000000			
Total	19	0.100710				

S	R-sq	R-sq (adj)	R-sq (pred)			
Model summary						
0.0003012	100.00%	100.00%	99.99%			

Significant values are in bold.

Similarly, the model of skin friction is provided by excluding the interaction variables in the manner directed in Table 9 (obtained from the ANOVA). As a result, a best-fitting model for the responses has been projected. This confidence in the model's ability to optimize and forecast has been established by the computed and adjusted values of 100%.

**Figure 14:**
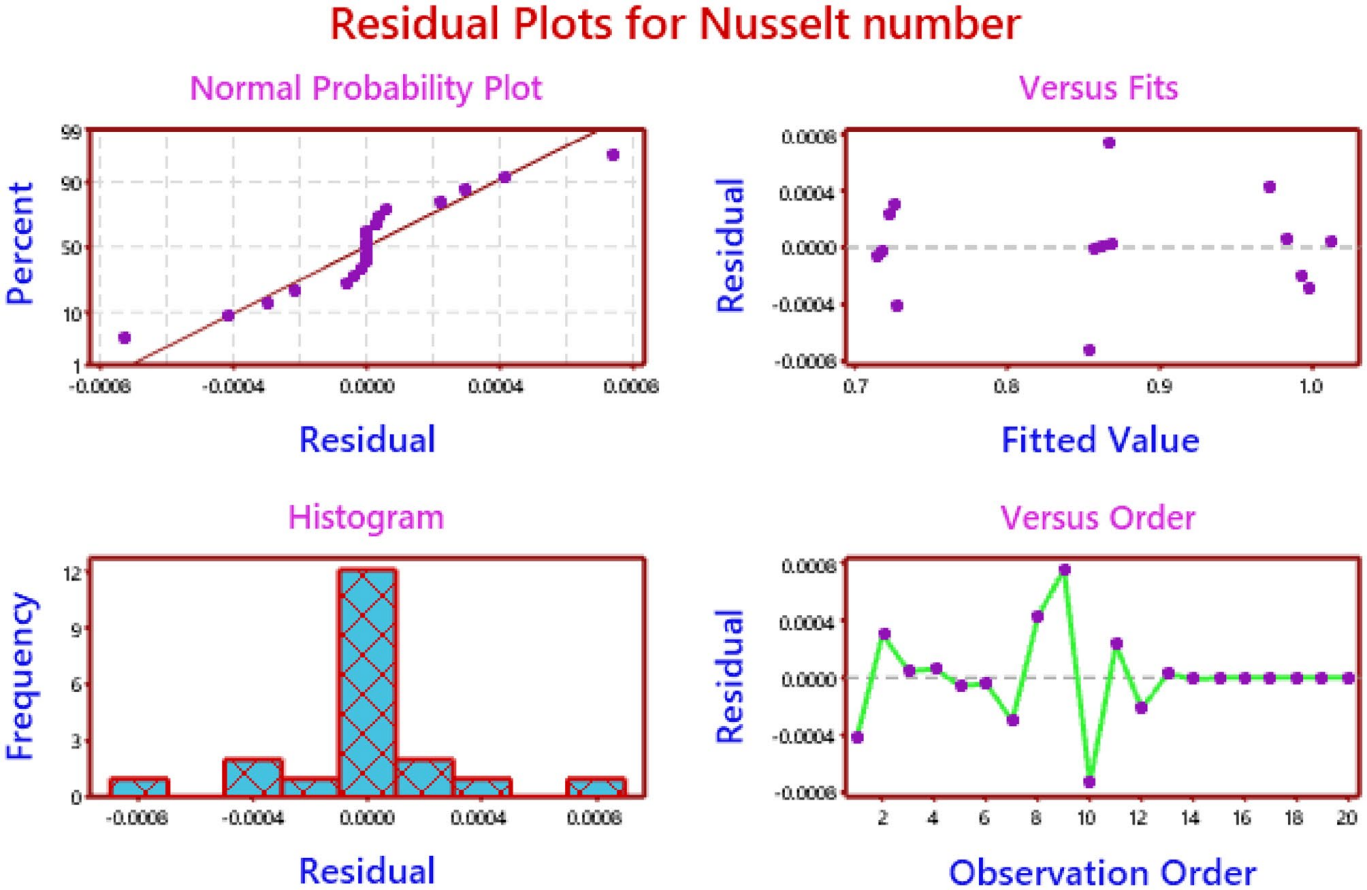
Residual plots for Nusselt number.

**Figure 15:**
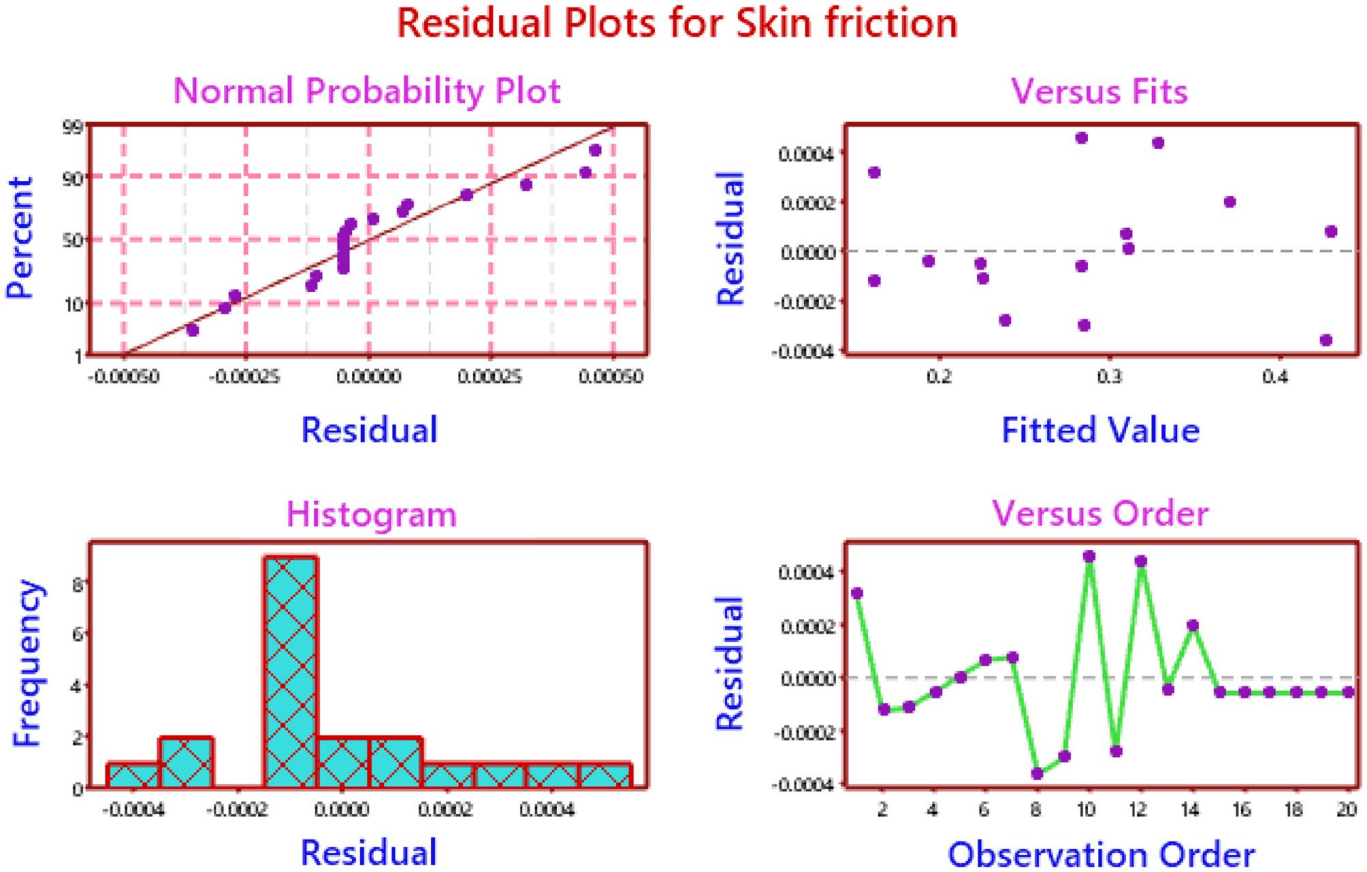
Residual plots for skin friction.

The normal plot for the distributions of the data with the fitting values is shown in Figs. 14 and 15, as well as the histogram for the same distribution and the order of the data distributions for the responses of Nusselt number and the skin friction coefficients, respectively. Due to the fact that the residuals are located close to the straight line, it is evident from these numbers that the model is appropriate.

**Figure 16:**
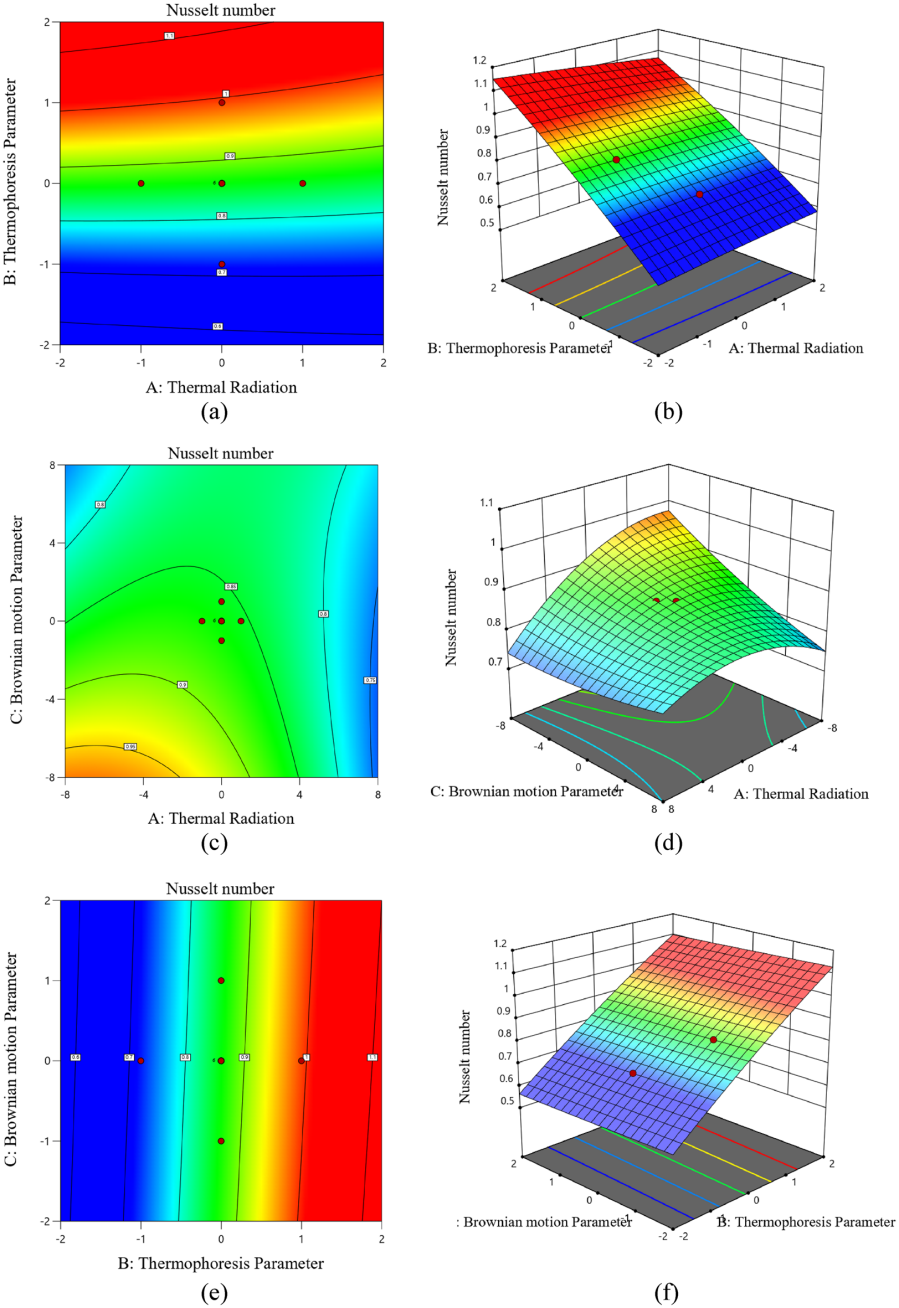
Contour (**a**, **c**, **e**) and surface (**b**, **d**, **f**) plots for $Nu_{x}$.

Figure 16a–f portrays the contour and surface plots for the interaction of two independent factors $\left( {Rd,Nb,Nt} \right)$ by maintaining one of them as the third factor at the middle level on the response of the heat transfer coefficient. The contour plots (a, c, and e) and the surface plots (b, d, and f) serve as examples of Nusselt number for various possible interactive factors. The contour lines and surface reflections in Fig. 16a,b respectively show that while Brownian motion is at middle level, the enhanced thermophoresis and lower level values of thermal radiation, the response function rises. Figure 16c,d illustrates the interaction factors' effect $Nb$ on the Nusselt number whenever $Nt$ maintained at the medium level. The result shows that the lower levels of two independent factors have significantly contributed to the enhancement in the response function.

**Figure 17:**
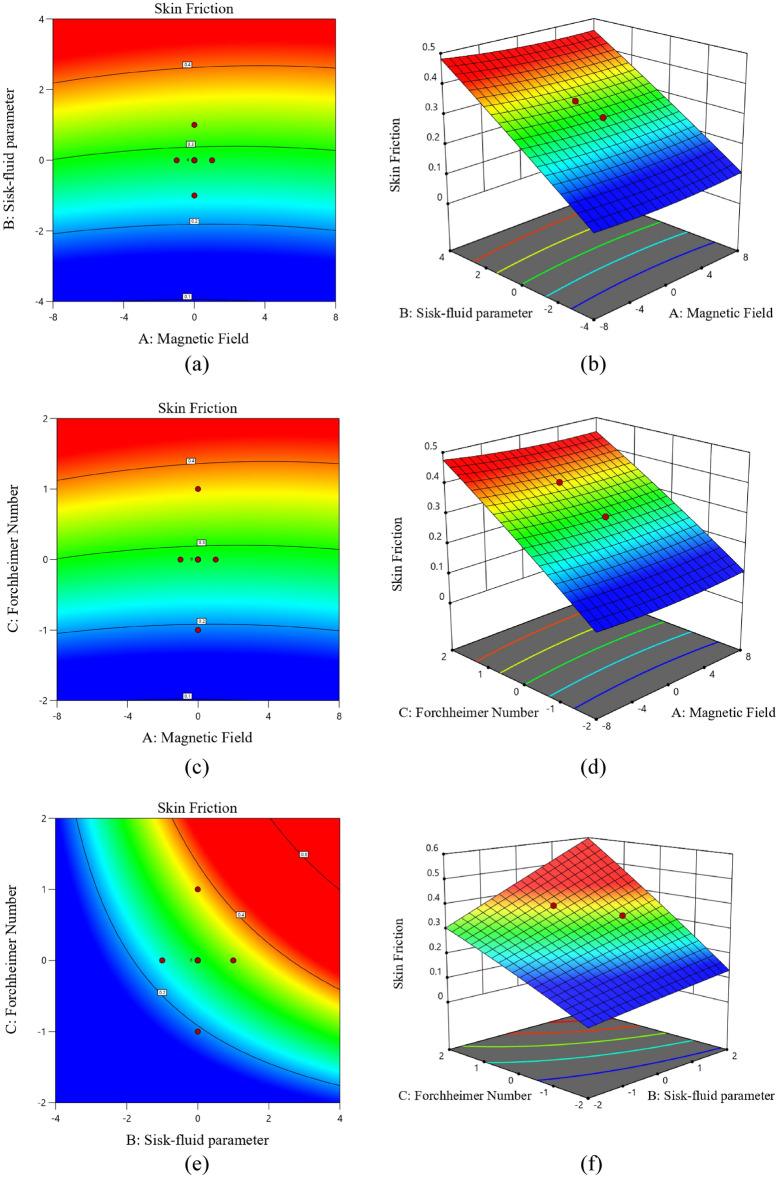
Contour (**a**, **c**, **e**) and surface (**b**, **d**, **f**) plots for $C_{f}$.

Furthermore, at a middle level of $Rd$ and $Nb$, saddle point is also witnessed on the surface of the response function. Figure 16e,f portrays the factors' role and the Nusselt number variation at the medium thermal radiation level $Rd$. The presented outcome reveals that the increasing $Nt$ and lower levels significantly enhance the heat transfer rate. Further, Fig. 17 depicts the interaction effects of the independent parameters $\left( {M_{1} ,\Lambda ,Fr} \right)$ on the variation of the skin friction coefficients considering one of them at the middle level. The responses are recorded through (a, c, e) contour plot and (b, d, f) the surface plots on different interactive factors. It is observed from Fig. 17a,b that the significant enhancement in the skin friction coefficient is marked with higher levels of, lower levels of, and medium levels of. Figure 17c,d exhibits the impact of $M_{1}$ and $Fr$ affects the skin friction coefficient rate. It is witnessed that the enhancement in the response function is observed at higher levels ofand lower level of magnetic field parameters. Figure 17e,f portrays the role of $\Lambda$ and $Fr$ on the skin friction coefficient enhancement while $M_{1}$ maintaining the middle level. The contours and surface depict that the skin friction coefficient is enhanced significantly for the increasing increase.

### Sensitivity analysis

Modeling and simulating the suggested design relies heavily on sensitivity analysis. The present study investigates the sensitivity of the heat transfer rate and the shear force rate to modest changes in parameters under controlled settings. It is defined as the partial derivative of the responses, which are the local Nusselt number and the shear rate, concerning the characterizing parameters $\left( {Rd,Nb,Nt} \right)$, which are referred to as coded variables. Calculating the partial derivative concerning the effective parameters looks like this:

$$\begin{aligned} & \frac{\partial Nu}{{\partial Rd}} = 0.006460 - 0.001009 *2*Rd - 0.006925*Nb \, + 0.001025*Nt \, \\ & \frac{\partial Nu}{{\partial Nb}} = 0.135520 - 0.004709*2*Nb \, - 0.006925*Rd \, - 0.000675 *Nt \\ & \frac{\partial Nu}{{\partial Nt}} = - 0.005830 + 0.001025*Rd \, - 0.000675 *Nb \\ \end{aligned}$$

and

$$\begin{aligned} & \frac{{\partial C_{f} }}{{\partial M_{1} }} = - 0.000680 - 0.000450*Fr \\ & \frac{{\partial C_{f} }}{{\partial \Lambda }} = 0.045290 + 0.013825*Fr \\ & \frac{{\partial C_{f} }}{{\partial Fr}} = 0.088680 - 0.001936*2*Fr - 0.000450*M{\mkern 1mu} + 0.013825*\Lambda \\ \end{aligned}$$

**Figure 18:**
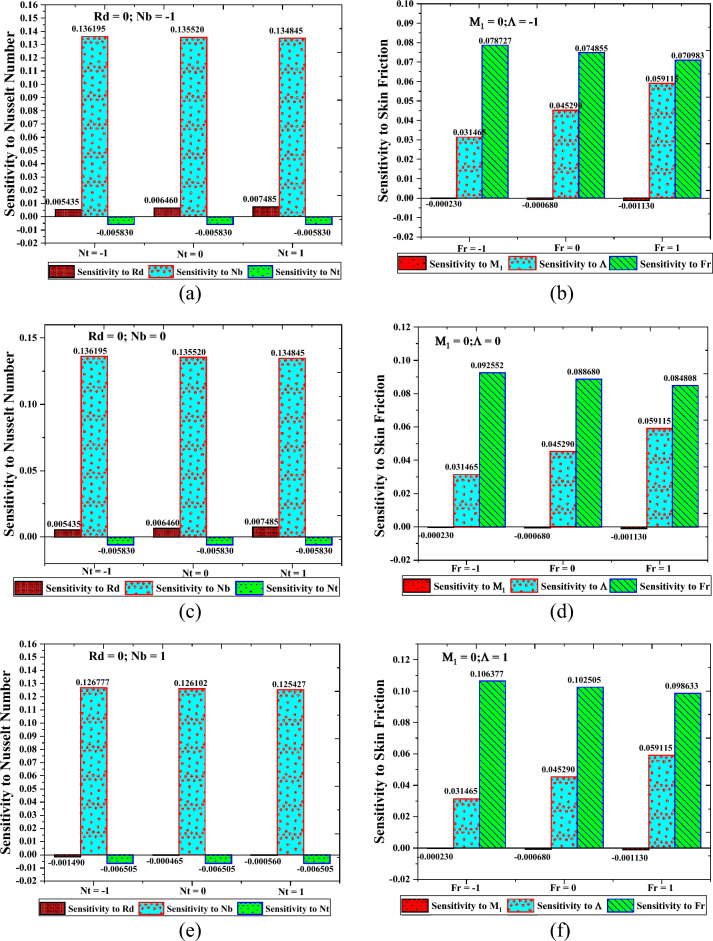
Sensitivity analysis of the Nusselt number (**a**, **c**, **e**) when $Rd = 0$ that is, at the middle level and for skin friction (**b**, **d**, **f**) when $M_{1} = 0$ that is, at the middle level.

Displayed here is the sensitivity of the rate of heat transfer as well as the friction factor in Fig. 18a,c,e and b,d,f correspondingly. The bar charts with both positive and negative values demonstrate how the surface friction and heat transfer rates have grown and reduced, respectively. From the figures, it can be observed that heat transport has the highest sensitivity value (0.145613) at the levels of $$\,(A = 0,B = - 1,C = - 1)$$$$Rd = 0.2,Nb = 0.4\,{\rm and}\, Nt = 0.1$$. The rise in the level of thermal radiation and the Brownian motion parameter both work in favor of the intensification of heat; however, the sensitivity towards thermophoresis at fixed levels $$Rd\,{\rm and}\, Nb$$ remains unchanged. At the same time, the highest sensitivity value (0.106377) of the rate of friction factor is observed at the levels of $$\,(A = 0,B = 1,C = - 1)$$$$M_{1} = 0.2,\Lambda = 0.5\,{\rm and}\, Fr = 0.2$$.

Similarly, the lowest sensitivity value (-0.00056) of heat transport is at the coded values of $Rd = 0.2,Nb = 0.4\,{\rm and}\, Nt = 0.1$, and the lowest sensitivity value (-0.00113) of surface friction is at the coded values of $M_{1} = 0.2,\Lambda = 0.1\,{\rm and}\, Fr = 0.6$. Furthermore, at the medium level of thermal radiation, the transport of heat sensitivity $(Rd,Nb,Nt)$ declined whenever the $Nb\,{\rm and}\, Nt$ increased from low-level to high-level values. It is also observed that the magnetic field parameter and Forchheimer parameter controls the rise in the sensitivity of the rate of friction factor $M_{1} \,{\rm and}\, Fr$. In contrast, an opposite trend is celebrated with the increase in the Sisk-fluid parameter.

## Conclusions

The current study provides numerical approximations for a steady, incompressible, two-dimensional Sisko-nanofluid flow on a stretching/shrinking surface equipped with power-law component (Sisko model), magnetic impact, thermal radiation, Brownian diffusion, and thermophoresis. In addition to boundary layers formulations, optimization analysis of the heat and mass transfer attributes is performed using Response Surface Methodology. The followings are the key outcomes of the study:

1. The influence of the involvement of porous medium in fluid flow analysis is prominent. Forchheimer number results in an inevitable decline in fluid flow. And so is the case with values of porosity parameter fluctuating between $0\le \lambda \le 2.5$. At $\lambda =0$, the fluid flow medium returns to a simple medium having no porosity and no additional frictional force.
2. Temperature distribution receives enhancement for larger thermal radiation, Brownian diffusion, and thermophoresis, which is quite logical from their constituency; however, a more significant Prandtl number results in a low thermal profile.
3. For both the cases, shrinking & stretching, we have noticed that the concentration of the particles shows specific rising behavior for the range $0\le {N}_{Th}\le 1,$ but the impact of thermophoresis becomes negligible close to 0. However, the results for both cases are the opposite of Brownian diffusion.
4. The involvement of Darcy-medium is quite helpful in controlling the fluid movement and balancing the fluid's thermal state for various manufacturing procedures while maintaining the nanofluid's low viscosity and higher thermal conductivity.
5. The coefficient of heat transfer is significantly achieved at higher levels of thermophoresis and lower levels of thermal radiation and Brownian motion factors. The surface friction rate at the plate boundary is dominant at higher values of Sisk-fluid parameter and Forchheimer parameter factors and lower levels of magnetic field parameter.
6. The transport of heat has the highest sensitivity value at the levels of $$\,(A = 0,B = - 1,C = - 1)$$$$Rd = 0.2,Nb = 0.4\,{\rm and}\, Nt = 0.1$$ while the highest sensitivity value of the rate of friction factor is observed at the levels of $$\,(A = 0,B = 1,C = - 1)$$$$M_{1} = 0.2,\Lambda = 0.5\,{\rm and}\, Fr = 0.2$$.
7. The transport of heat sensitivity $(Rd,Nb,Nt)$ declined whenever the $Nb\,{\rm and}\, Nt$ increased from low to high and at the medium level of thermal radiation. Increasing the magnetic field parameter and the Forchheimer parameter increases the sensitivity of the rate of friction factor, whereas increasing the Sisk-fluid parameter has the reverse effect.

## Data Availability

All data generated or analyzed during this study are included in this published article.
